# How I Treat: Haploinsufficiency of A20

**DOI:** 10.70962/jhi.20250138

**Published:** 2026-05-04

**Authors:** Jonathan Li, Jeffrey Y. Zhou, Manuel Carpio Tumba, Tingyan He, Kader Cetin Gedik, Daniella M. Schwartz

**Affiliations:** 1 Division of Rheumatology and Clinical Immunology, University of Pittsburgh Medical Center, Pittsburgh, PA, USA; 2 Division of General Internal Medicine, University of Pittsburgh Medical Center, Pittsburgh, PA, USA; 3 Autoinflammatory Center of Excellence, University of Pittsburgh Medical Center, Pittsburgh, PA, USA; 4 Pittsburgh Immunogenetics Discovery Center, University of Pittsburgh Medical Center, Pittsburgh, PA, USA; 5 Division of Rheumatology and Immunology, Shenzhen Children’s Hospital, Shenzhen, China; 6 Division of Pediatric Rheumatology, University of Pittsburgh Medical Center, Pittsburgh, PA, USA

## Abstract

Haploinsufficiency of A20 (HA20) is a primary immune regulation disease caused by heterozygous loss-of-function variants in *TNFAIP3*, resulting in unchecked inflammatory signaling. HA20 is a highly heterogeneous disorder with overlapping features of autoinflammation, autoimmunity, immunodeficiency, atopy, and lymphoproliferation. Most patients develop symptoms in early childhood mimicking Behcet’s disease, inflammatory bowel disease, periodic fevers with aphthous stomatitis, pharyngitis, and adenopathy (PFAPA), systemic lupus erythematosus (SLE), autoimmune hepatitis, vasculitis, and other conditions. This phenotypic variability contributes to diagnostic delays. Diagnosis requires identification of a pathogenic *TNFAIP3* variant or deletion encompassing *TNFAIP3*: most are null, but missense variant interpretation remains challenging. After diagnosis, we use comprehensive clinical laboratory studies, imaging, and multidisciplinary evaluations to screen for complications. Treatment is guided by clinical phenotype and biomarkers. Colchicine and PDE4 inhibitors may control mild disease, whereas IL-1, TNF, and JAK inhibitors are often necessary for moderate-severe cases. Acute withdrawal of immunosuppression can precipitate disease flares. Genetic counseling and evaluation of at-risk family members are essential.

## Teaser

Li et al. present a comprehensive clinical framework for diagnosing and managing HA20, an immune dysregulation disease caused by loss-of-function TNFAIP3 variants. The authors outline clinical evaluation strategies, family screening, and phenotype- and biomarker-guided treatment strategies using IL-1, TNF, and JAK inhibitors.

## Introduction

Haploinsufficiency of A20 (HA20) is an autosomal-dominant immune dysregulation disease first described in 2016 (1). Prior to this, A20 variants had been identified as risk alleles for various autoimmune and autoinflammatory diseases (e.g., inflammatory bowel disease [IBD], rheumatoid arthritis, systemic lupus erythematosus (SLE), and type 1 diabetes) (2). A20 is a negative regulator of inflammation encoded by the tumor necrosis factor α-induced protein 3 gene (*TNFAIP3*). Heterozygous loss-of-function *TNFAIP3* variants therefore result in unchecked immune activation initially described as a predominantly Behcet’s-like systemic autoinflammatory disease (SAID). However, given the diverse roles of A20 in immune function, autoimmunity, immunodeficiency, atopy, and malignancy are also common among patients with HA20 (3, 4). Even within families, there is marked heterogeneity, and no definitive genotype–phenotype associations have been recognized so far (5). This disease heterogeneity inherently causes diagnostic delays, with molecular genetic testing at a median of 7 years after symptom onset (4).

The true prevalence of HA20 is unknown, but to date ∼200 cases have been reported worldwide, linked to about 100 distinct pathogenic variants. Analysis of population databases indicates the collective prevalence of individually rare (minor allele frequency [MAF] <0.01%) null *TNFAIP3* variants, which are classified as “pathogenic very strong” (PVS1) by American College of Medical Genetics (ACMG), Bethesda, MD, USA, standards, may be as high as ∼1:14,000. This suggests that ∼500,000 individuals may carry disease-causal *TNFAIP3* variants (6). Hence, HA20 is likely vastly underrecognized despite clear implications for disease monitoring and treatment. Given the paucity of validated clinical criteria and guidelines for diagnosing, monitoring, or treating HA20, this review aims to highlight key principles in our approach to patients with known or suspected HA20.

## Functions of A20: A brief overview

A20 is encoded by the *TNFAIP3* gene on chromosome 6 (2). In its fully translated form, A20 is a 790–amino acid ubiquitin-editing enzyme that modifies polyubiquitin chains on various immune signaling proteins. Structurally, the protein is composed of an ovarian tumor (OTU) domain and seven zinc-finger (ZnF) domains, which collectively mediate ubiquitin editing. In most cell types, A20 is minimally expressed in the resting homeostatic state but is induced during immune activation, functioning as an essential negative regulator of inflammation (7).

A20 exerts its ubiquitin-editing functions via coordinated activity of its OTU and ZnF domains. The N-terminal OTU domain removes activating K63-linked polyubiquitin chains from signaling adaptors and is required for the recruitment of E2 enzymes like Ubc13 and UbcH5c (8). The ZnF4 domain is a scaffold that supports K48-linked ubiquitination and proteasomal degradation of inflammatory signaling molecules via interactions with adaptor proteins (e.g., TAX1BP1) and E3 ligases (e.g., Itch and RNF11) (9). A20 does not directly interact with E2 enzymes but is required for binding to key substrates (10). The C-terminal ZnF7 domain binds M1-linked (linear) ubiquitin chains, recruiting A20 to the TNF signaling complex, where it disrupts NEMO–LUBAC interactions to repress NF-κB activation (11). A20 also has non-catalytic interactions with binding partners (e.g., NEMO and TRAF2) that can disrupt E2–E3 enzyme complexes, which is an additional mechanism that terminates inflammatory signaling (8).

**Figure 1.**
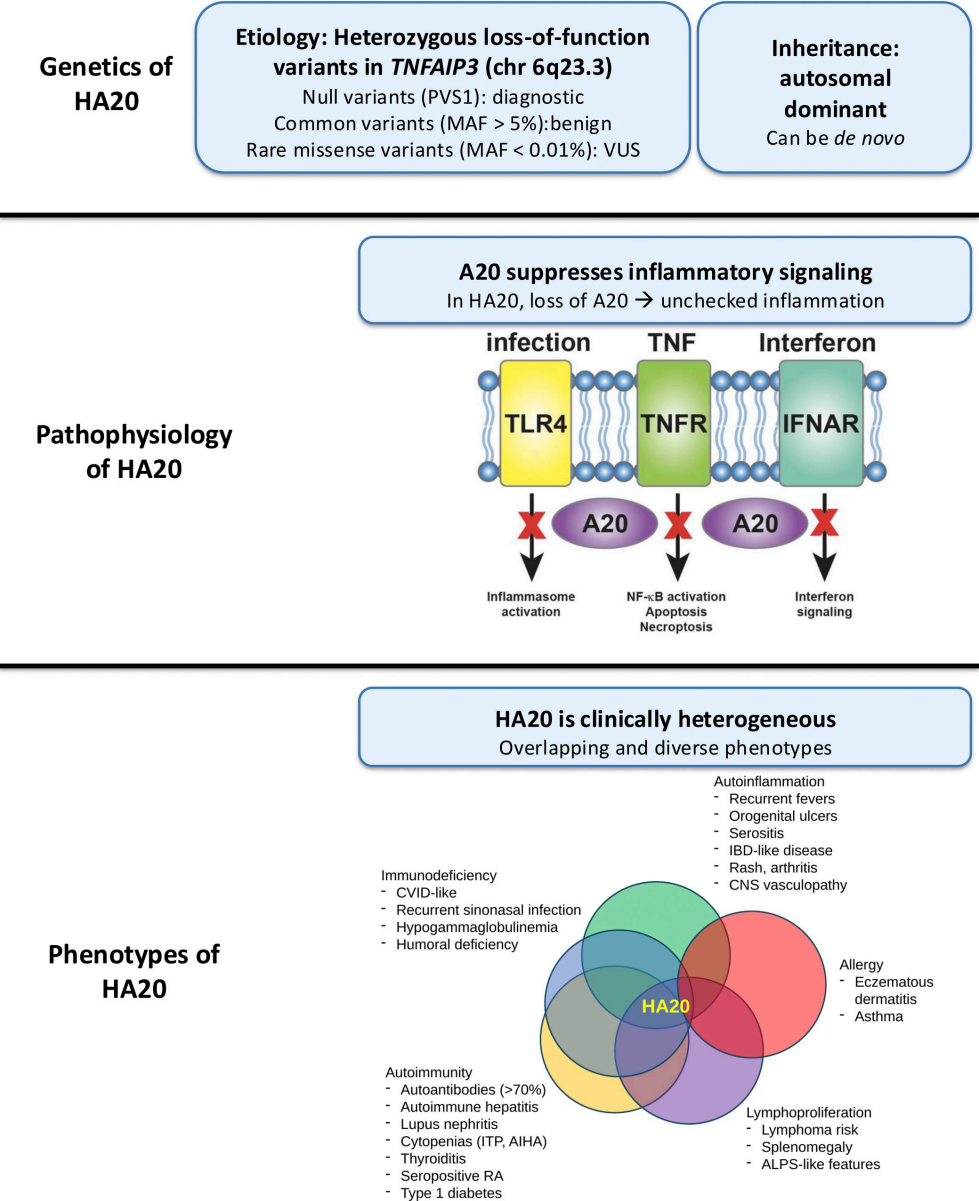
**HA20: Etiology, pathophysiology, and clinical phenotypes.** Schematic presents a broad overview covering the genetic mechanism of HA20 (loss-of-function heterozygous variants in *TNFAIP3*), the immunology, and pathophysiology, including major pathways implicated in the immune dysregulation of HA20 and a selected list of HA20-associated phenotypes. PVS1, pathogenic very strong 1; TLR4, Toll-like receptor 4; TNFR, tumor necrosis factor receptor; NF-κB, nuclear factor κ-B; IFN, interferon; CVID, common variable immunodeficiency; ITP, immune thrombocytopenic purpura; AIHA, autoimmune hemolytic anemia; RA, rheumatoid arthritis; ALPS, autoimmune lymphoproliferative syndrome.

A20 is best characterized as a negative regulator of NF-κB signaling (Fig. 1) (7, 12). Loss of A20 renders cells hypersensitive to various inflammatory stimuli, resulting in increased production of NF-κB–induced cytokines, including TNF-α, IL-1, IL-6, and IL-8. A20 also regulates NLRP3 inflammasome activity and related processes like necroptosis (13, 14, 15). Finally, A20 may influence interferon signaling pathways by modulating STAT1 levels and pathogen double-stranded RNA sensing (16, 17, 18, 19).

As expected from these multifunctional roles, loss of A20 results in global immune dysregulation, including autoinflammation, autoimmunity, immunodeficiency, atopy, and malignancy (Fig. 1). Most reported *TNFAIP3* variants in patients are predicted loss of function (e.g., stop-gained and frameshift); missense mutations are also reported to cause HA20, but data for some variants have been contradictory, and better assays are needed to diagnose HA20 in patients with potentially deleterious missense variants (4).

## Clinical presentation: When to suspect HA20?

HA20 was first described in 2016 in a case series of six unrelated families with Behcet’s disease (BD)–like features (1). Since then, epidemiologic studies and case reports have demonstrated significant clinical heterogeneity (4, 20, 21). Because HA20 was initially described as a BD-like disease, current reports likely overrepresent patients with BD-like clinical features, as these individuals are more frequently selected for *TNFAIP3* testing. Table 1 summarizes reported disease features using data from three published reviews (3, 4, 21) and from an international multicenter cohort from our group describing 185 cases assessed longitudinally in 41 clinics and seven countries (22). Some overlap exists between cohorts.

**Table 1.** Major reported clinical phenotypes of HA20

​	Karri et al. 2024 (3)	Elhani et al. 2024 (4)	Shiraki et al. 2025 (21)	Others (21, 23, 24, 25, 26);	Multicenter cohort (27)
**Demographics**	​	​	​	​	​
# of patients	199	177	54	12	185
# of families	130	NR	37	11	112
Population	East Asia, Turkey, Europe, and United States	East Asia, Turkey, Europe, and United States	Japan and Vietnam	East Asia and Europe	United States, Asia, Europe, South and Central America, and New Zealand
Age of onset, years	Mean 7 (0–39)	Median 4 (0–35)	Median 2.8 (0–27), 60% before age 5	Mean 4.8 (0–26) *N* = 10	Median 3.3 (0.04–33)
Female	NR	62%	54%	58.3%	55.2%
**Autoinflammation**	​	​	​	​	​
Oral ulcers	68%	69%	78%	75%	72.2%
Genital ulcers	37%	36%	35%	42%	32%
Rash	42%	43%	50%	42%	43.8%
Arthritis/arthralgia	34%	31%	37%	50%	46.7%
Ocular	8.5%	8%	11%	17%	6%
Gastrointestinal, IBD-like	39%	46%	76%	58%	58.6% (27.7% with intestinal ulcers)
Vascular disease	11%	8%	4%	17%	NR
Neurologic disease	11.5%	10%	18.5%	8%	16.6%
Pathergy	1.5%	NR	NR	NR	NR
Recurrent fever	49%	54%	85%	58%	63.3%
Lung disease	+	6%	6%	NR	20.2%
HLH/cytokine storm	+	NR	+	NR	NR
**Autoimmunity**	​	​	​	​	​
Autoantibodies	72%	NR	NR (all autoimmunity 28%)	83%	ANA 37.2%, antithyroid antibodies 15.9%, Coombs 25%, and other autoantibodies 35.9%
Thyroid	14%	14%	17%	17%	17.6%
T1DM	4%	3%	2%	17%	+
Liver disease	14.5%	10%	15%	17%	28.9%
SLE	7%	9%	6%	NR	+
Cytopenias	20%	6%	7%	42%	56.6%
**Lymphoproliferation**					
ALPS-like	1%	NR	NR	NR	NR
Lymphoma	1%	NR	2%	NR	+
Lymphadenopathy	16%	NR	15%	NR	32%
**Immunodeficiency**					
CVID	1.5%	All immunodeficiency 5%	NR	NR	+
Humoral deficiency	5.5%		NR	8%	+
Recurrent infection	9%		NR	NR	35.5%

NR, not reported; HLH, hemophagocytic lymphohistiocytosis; T1DM, type 1 diabetes mellitus; ALPS, autoimmune lymphoproliferative syndrome; CVID, common variable immunodeficiency; +, reported but frequency not known.

Most patients with HA20 develop symptoms before age 10 (median 3–4 years) and as early as infancy. The most common symptoms include orogenital ulcers, recurrent fever, rash, arthritis, lymphadenopathy, IBD-like symptoms, and autoimmunity. Primary immune regulation disease (PIRD) should be suspected among patients with early childhood onset presentations, unusually refractory or severe disease, or accrual of seemingly unrelated immunologic diagnoses over time. Categorizing diagnoses by immunopathogenic endotype (autoinflammatory, autoimmune, atopic, lymphoproliferative, immunodeficient, and nonimmune) can help identify and stratify potential PIRDs like HA20. For these reasons, a detailed history is essential in calibrating suspicion for HA20. History should begin with the prenatal course, infancy, and childhood, with attention to growth and development, infection frequency and severity, and vaccine reactions in addition to typical symptoms like recurrent fevers or oral ulcers. Once HA20 is suspected, a detailed family history should be obtained seeking an autosomal-dominant pattern of immune dysregulation. Because de novo germline mutations can also cause HA20, a negative family history should not preclude genetic testing.

## Diagnosis/genetic testing

**Figure 2.**
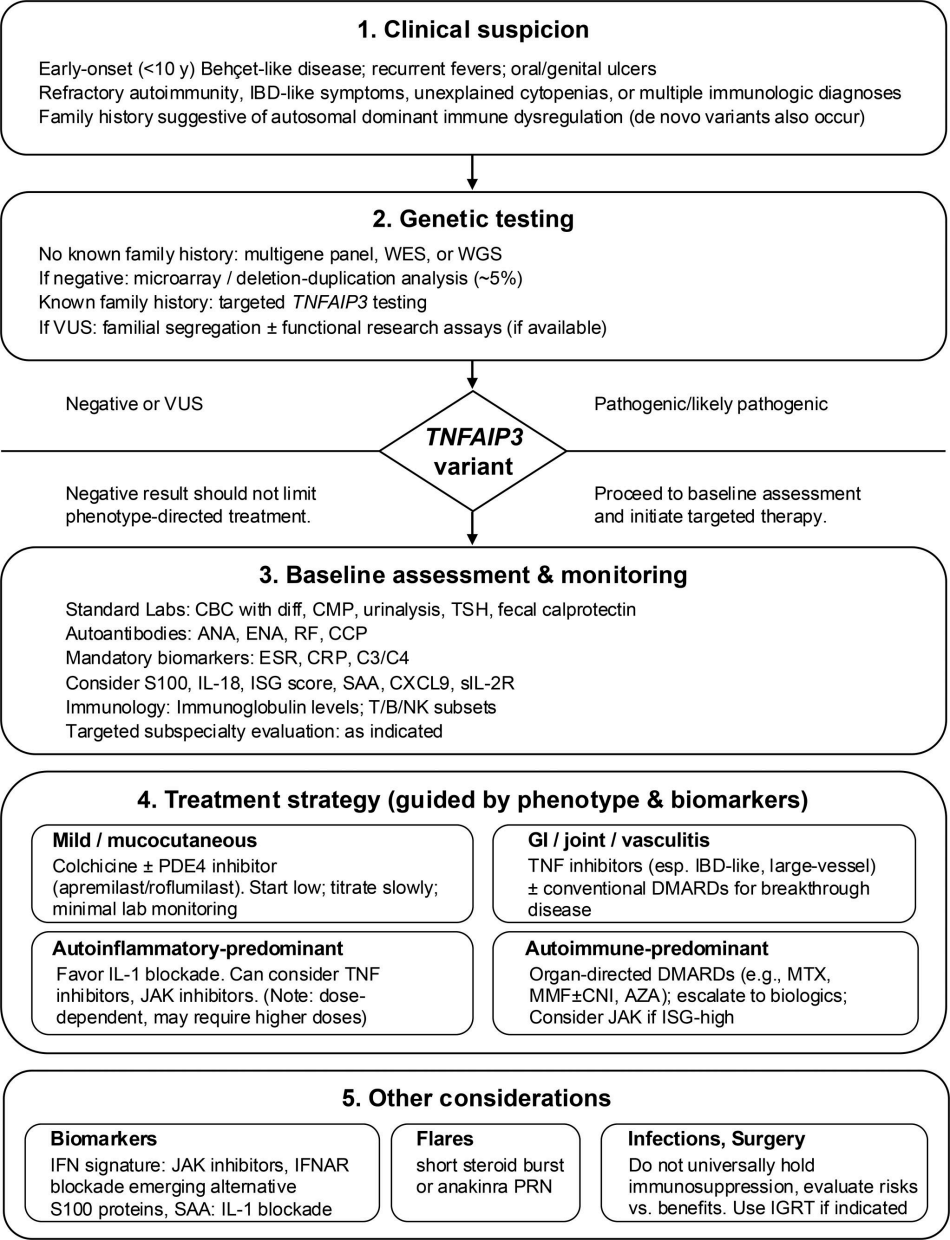
**Diagnostic and therapeutic algorithm for HA20.** Flowchart presents a proposed algorithm outlining our diagnostic and therapeutic approach for patients with suspected HA20. This includes symptoms prompting clinical suspicion (classical and atypical phenotypes), options for genetic and functional testing, baseline and serial clinical studies for assessment and monitoring, and other considerations. WES, whole-exome sequencing; WGS, whole-genome sequencing; CBC with diff, complete blood count with differential; CMP, complete/comprehensive metabolic panel; TSH, thyroid-stimulating hormone; ANA, anti-nuclear antibody; ENA, extractable nuclear antigen antibody; RF, rheumatoid factor; CCP, cyclic citrullinated peptide; ESR, erythrocyte sedimentation rate; CRP, C reactive protein; C3/C4, complement 3/complement 4; IL-, interleukin; SAA, serum amyloid A; CXCL9, CXC motif chemokine ligand 9; PDE4, phosphodiesterase 4; GI, gastrointestinal; TNF, tumor necrosis factor; JAK, Janus kinase; MTX, methotrexate; MMF, mycophenolate mofetil/mycophenolic acid; CNI, calcineurin inhibitor; AZA, azathioprine; PRN, pro re nata (as needed); IGRT, immunoglobulin replacement therapy.

HA20 is a genetically defined disease: as such, the diagnosis should not be made based on a compatible clinical history without confirmatory genetic testing (Fig. 2). Because the phenotypic spectrum of HA20 is broad and can overlap with other inborn errors of immunity (IEI), we test patients without a known family history of HA20 for a broad group of IEI using a molecular multigene panel, whole-exome sequencing, or whole-genome sequencing. This simultaneously assesses *TNFAIP3* and >500 other genes known to cause IEI (28). By contrast, if patients have a known family history of HA20, we only test for *TNFAIP3.* A diagnosis of HA20 is made when a heterozygous pathogenic variant of *TNFAIP3* or a heterozygous deletion of chromosome 6 containing the *TNFAIP3* gene is identified. Approximately 5% of variants are identified using chromosomal microarray analysis or gene-targeted deletion/duplication analysis, so these should be considered for patients with normal sequence analysis (29). Although a pathogenic variant in *TNFAIP3* is required for a definitive diagnosis of HA20, negative genetic testing results should not limit treatment.

## Missense variants

Most known pathogenic variants in *TNFAIP3* are null (nonsense, frameshift, complete deletions, and splice null). These are classified as PVS1 by ACMG guidelines and should be considered diagnostic. By contrast, common missense variants (MAF >5%) are associated with increased autoimmune disease risk but should not be considered diagnostic of HA20. Rare (MAF <0.01%) pathogenic *TNFAIP3* missense variants have been described but can be difficult to interpret without functional research assays (3). Accordingly, ∼80% of rare missense variants are designated variants of unknown significance (VUS). Interpretation of VUS should incorporate allele frequency, suspected impact on protein structure (based on in silico modeling, e.g., Revel and AlphaMissense), prior reports, functional assays, genotype–phenotype segregation within families, and other factors (30).

To better characterize VUS, we routinely perform familial variant testing and functional analysis at the bench. The “gold standard” assay requires overexpression of wild-type (WT) vs. mutant *TNFAIP3* constructs in cell lines, followed by stimulation with TNF-α and measurement of NF-κB activation via luciferase activity, P65 phosphorylation and/or nuclear localization, IκBα degradation, or IKKα/β phosphorylation (1, 27, 31, 32). WT A20 and ACMG benign (BS3) variants suppress NF-κB activation, whereas null variants fail to do so. Other modalities include measurement of TNF-induced NF-κB activation or basal A20 levels in patient-derived cells (e.g., monocytes and dermal fibroblasts) or of mutant A20 protein stability in overexpression assays (1, 32, 33, 34). Because ACMG “pathogenic strong” (PS3) classification requires functional studies in isogenic systems (35), we test all coding variants in cell lines. However, overexpression assays have important limitations, including inability to evaluate noncoding or splice variants, overexpression artifact, missing regulatory mechanisms (e.g., splicing defects, posttranslational modification, and degradation), and absent cellular context in nonimmune cells. Additionally, A20 regulates pathways beyond TNF–NF-κB signaling, including inflammasome activation, interferon signaling, and cell death. For missense variants with inconclusive results in isogenic systems, we therefore measure basal A20 expression and TNF-induced NF-κB activation in primary patient cells; in some cases, we measure effects on other A20-regulated pathways. Nonetheless, results for some variants remain inconclusive even after extensive testing, and these assays are not broadly available to clinicians. Functional data for some *TNFAIP3* variants can be found on the Infevers database (36), and ClinGen curations for TNFAIP3 are in development.

## Clinical workup and monitoring

Once HA20 is diagnosed, we obtain a baseline panel of clinical laboratory testing and imaging studies to identify features of immune dysregulation, assess disease activity, and screen for HA20-related morbidities (Fig. 2 and Table 2). These include thyroiditis, hepatitis, ocular disease, SLE-like nephritis, central nervous system (CNS) vasculopathy or neuroinflammation, coronary vasculopathy, lymphoma, and IBD-like disease. When possible, we obtain disease activity biomarkers both during noninfectious flares and during intercritical periods, as these can differ substantially. Disease activity biomarkers should not be obtained during infection, as they may be nonspecifically elevated.

**Table 2.** Baseline and annual evaluations

System	Baseline	Routine monitoring	Situational
Rheumatologic	Autoantibodes (ANA, Sm, RNP, SSA, SSB, APL Abs ANCA, RF, and CCP) Mandatory disease activity markers^a^: ESR, CRP, C3/C4 Facultative disease activity markers^a^: S100A8/9, S100A12, SAA. CXCL9, IL-18, sIL2R, IFN signature (nanostring, four-gene panel, monocyte CD169/CD274)	At least annually: Mandatory disease activity markers^a^: ESR, CRP, and C3/C4 Facultative disease activity markers^a^: S100A8/9, S100A12, SAA, IL18, CXCL9, sIL2R, and IFN signature (nanostring, four-gene panel, monocyte CD169/CD274)	Plasma cytokines Imaging of affected joints CK, aldolase Additional autoantibodies (myositis, scleroderma related)
Dermatologic	Skin exam and symptom screen by primary provider	At least annually: Skin exam, symptom screen	Skin biopsy
Dental/oral health	Symptom screen	At least annually: Symptom screen	Salivary flow measurement Salivary gland imaging MSG biopsy Dental evaluation (unusual caries)
Neurologic	Symptom screen	At least annually: Symptom screen	CNS: Brain MRI, MRA, MRV, and LP PNS: EMG ± biopsy, comprehensive neuropsychiatric testing
Ophthalmologic	Comprehensive eye exam, slit lamp, retinal, tear film staining	At least annually: Comprehensive eye exam	Uveitis, scleritis, episcleritis, vasculitis, and dry eye disease
Cardiac	Symptom screen	At least annually: Symptom screen	Myocarditis: Cardiac MRI, cardiac PET, cardiac catheterization, cardiac enzymes, lipids, TTE, and EKG
Pulmonary	Symptom screen	At least annually: Symptom screen	PFTs, CXR, HRCT chest, bronchoscopy, and VATS
Gastrointestinal	(In addition to general tests) Hepatic panel, fecal calprotectin Fib4 score	At least annually: Hepatic panel, Fib4 score As needed: Calprotectin	Micronutrient levels EGD Enteroscopy Colonoscopy Abdominal imaging (CT and US) Autoimmune hepatitis serologies Celiac screen
Renal	(In addition to general tests) BMP (serum electrolytes, BUN, and Cr) UA and UPCR	At least annually: UA, UPCR, and BMP	If concern for CKD: Renal US and kidney biopsy
Endocrine	Growth curves, TSH, free T4 Antithyroid Abs A1C	At least annually: Growth curves (for children), TSH, free T4 Situational: A1c, fasting glucose, and antithyroid Abs	DEXA scan T1DM-related autoantibodies
Hematologic and oncologic	CBC with differential, PT/PTT, iron, and B12 APL Abs, cancer screening	At least annually: CBC with diff, cancer screening (age appropriate) As needed: PT/PTT, Iron, B12 APL Abs	Anti-IF antibodies Anti-PLT antibodies DAT, Coombs SPEP, UPEP, IEP, and K/L FLC Bone marrow biopsy, PET-CT
Allergic and immunologic	Quantitative immunoglobulins T/B/NK subsets Vaccine titers Annual vaccinations	At least annually: Quantitative immunoglobulins, T/B/NK subsets Annual vaccinations As needed: Vaccine titers	IgG subsets B cell subsets T cell subsets Anti-cytokine antibodies

ANA, antinuclear antibody; Sm, Smith; RNP, ribonucleoprotein; APL, antiphospholipid; ANCA, antineutrophil cytoplasmic antibody; RF, rheumatoid factor; CCP, cyclic citrullinated peptide; ESR, erythrocyte sedimentation rate; CRP, C-reactive protein; SAA, serum amyloid A; IL, interleukin; sIL2R, soluble IL-2 receptor; IFN, interferon; CK, creatine kinase; MSG, minor salivary gland; MRI, magnetic resonance imaging; MRA, magnetic resonance angiography; MRV, magnetic resonance venography; LP, lumbar puncture; EMG, electromyography; PET, positron emission tomography; TTE, transthoracic echocardiogram; EKG, electrocardiogram; PFT, pulmonary function test; CXR, chest x-ray; HRCT, high resolution computed tomography; VATS, video assisted thoracoscopic surgery; EGD, esophagogastroduodenoscopy; US, ultrasound; BMP, basic metabolic panel; BUN, blood urea nitrogen; Cr, creatinine; UA, urinalysis; UPCR, urine protein:creatinine ratio; CKD, chronic kidney disease; TSH, thyroid-stimulating hormone; DEXA, dual energy x-ray absorptiometry; T1DM, type 1 diabetes mellitus; CBC, complete blood count; PT, prothrombin time; PTT, partial thromboplastin time; IF, intrinsic factor; PLT, platelet; DAT, direct antigen test; SPEP, serum protein electrophoresis; UPEP, urine protein electrophoresis; IEP, immunoelectrophoresis; K/L FLC, κ/λ-free light chain; IgG, immunoglobulin G.

a Mandatory vs. facultative disease activity markers: We recommend checking broadly available disease activity markers (labeled “mandatory”) for all patients with HA20. Other disease activity markers (labeled “facultative”) may be available only at selected diagnostic immunology labs. However, we have seen patients with active organ-threatening disease (i.e., liver disease and neuroinflammation) who have normal standard disease activity markers but elevated facultative disease activity markers. We therefore check both mandatory and facultative markers annually in all patients.

Baseline tests obtained in all patients include general laboratory assessments (e.g., complete blood counts with differential to screen for cytopenias, comprehensive metabolic panel, and urinalysis); and acute phase reactants (C-reactive protein, erythrocyte sedimentation rate, and complement [C3/C4] levels). If possible, we also obtain immunologic disease activity biomarkers to quantify the severity and nature of inflammation. Several disease activity markers (e.g., S100 proteins and interferon-stimulated gene [ISG] score) may not be widely available, especially in resource-limited settings. Patients in remission or with minimal disease activity may be able to forego these studies. However, we have observed patients with normal acute phase reactants who experience continued disease-related symptoms and even end-organ damage in the setting of elevated ISG score and/or S100 proteins. We therefore maintain a low threshold for ordering these more targeted assays; when they are not available at the time of assessment, we recommend banking patient-derived samples for future measurements.

In addition to immunologic biomarkers, we obtain subspecialist-level examinations for organ-threatening and life-threatening morbidities (e.g., slit lamp exam and tear film staining for ocular involvement). Fecal calprotectin and thyroid stimulating hormone are tested annually to assess for developing IBD or thyroid disease; in patients with abdominal symptoms, we obtain endoscopy/colonoscopy and/or abdominal imaging. More than 70% of HA20 patients develop autoantibodies, including patients with an autoimmunity-predominant phenotype who do not have appreciable acute phase reactant changes; we therefore test for autoantibodies (e.g., antinuclear antibody, antibodies to extractable nuclear antigens, rheumatoid factor, and cyclic citrullinated peptide) in all patients. We obtain radiographic studies for patients with significant arthritis and dedicated cardiac, pulmonary, and/or neurologic evaluations for patients with significant symptoms. Immune deficiencies are present in nearly 25% of patients in our cohort, and we therefore obtain quantitative immunoglobulin levels and T/B/natural killer (NK) subset levels at baseline.

We recommend that all patients maintain a close relationship with both their primary care physician and their rheumatologist as medical homes. Well-controlled patients see us and other relevant subspecialists for routine monitoring, including measurement of disease activity markers and screening for developing asymptomatic end-organ involvement (e.g., liver or kidney disease). Patients with ongoing active disease, whether clinical or subclinical, are seen and monitored more frequently. When monitoring patients, we maintain a high awareness for inflammatory, immunologic, and oncologic complications.

## Treatment of HA20

Although there are no Food and Drug Administration (FDA)–approved treatments or guidelines for HA20, empiric treatment can prevent morbidity and improve quality of life. Many conventional and biologic disease-modifying antirheumatic drugs (DMARDs) have been used in HA20, likely reflecting the disease’s substantial phenotypic and molecular heterogeneity (Table S1). The most used agents are corticosteroids, colchicine, TNF inhibitors, IL-1 inhibitors, and JAK inhibitors.

Some patients with autoinflammatory-predominant features (e.g., recurrent fevers, serositis, mucosal disease, and elevated acute phase reactants) and mild disease can achieve remission with colchicine monotherapy. Patients with mucosal disease may respond to PDE4 inhibitor, including apremilast, which is FDA-approved for BD. The cheaper generic PDE4 inhibitor roflumilast is not approved for BD but has shown efficacy in small studies (37, 38, 39). These can be combined with colchicine with relatively low risk and minimal laboratory monitoring. To minimize gastrointestinal adverse effects, both colchicine and PDE4 inhibitors should be initiated at a low dose and titrated slowly. Some reports suggest colchicine may be associated with lactose intolerance, so patients could consider limiting dairy or using lactase supplements (40).

For patients who fail these relatively well-tolerated, nonspecific therapies or have severe, organ-threatening disease, we use the clinical phenotype and biomarker profiles to guide selection of targeted therapies. For example, patients with elevated markers of inflammasome-related myeloid activation (IL-18, S100 proteins) or serum amyloid A are more likely to be treated with IL-1–targeted therapy as a first-line strategy. Conversely, we often treat patients with elevation of type 1 ISGs with JAK inhibitors (41). Once therapy is initiated, trending biomarkers can also help assess treatment response, with interpretation guided by clinical improvement. If biomarkers are broadly elevated, clinical features may remain central to therapeutic selection. Similarly, if biomarker assessments are not readily available (e.g., in consultations from outside centers and other countries), we base treatment decisions on clinical presentation, particularly the presence and extent of internal organ involvement.

In patients with autoinflammatory-predominant disease who fail colchicine or who have severe inflammation, we generally start with IL-1 inhibition due to the favorable adverse effect profile and favorable outcomes (22). The IL-1 receptor antagonist anakinra has a rapid onset and short half-life: patients can be started at 1–2 mg/kg daily and dose titrated up to 5–8 mg/kg/day as for other autoinflammatory conditions (42, 43). However, daily injections may be challenging, and site reactions can become severe at higher doses. With long-term use, concerns have also been raised about cutaneous or systemic amyloidosis (44, 45). We therefore often transition patients from anakinra to canakinumab as a long-term maintenance regimen with a lower injection burden. Nonetheless, some patients report superior responses to anakinra, particularly those with CNS disease and severe mucocutaneous disease (46). For severe mucocutaneous disease, we also maximize colchicine therapy and add a PDE4 inhibitor. Rilonacept can be a useful alternative to anakinra in this setting; however, due to competing mechanisms of action, rilonacept cannot be used in combination with anakinra (47). For patients with CNS involvement, combination therapy with anakinra (at lower doses) and canakinumab may be required.

Alternative strategies for autoinflammation-predominant disease include TNF inhibition and JAK inhibition (1, 3, 41). TNF inhibition is particularly useful for gastrointestinal and joint disease, and we have also successfully used TNF inhibitors in patients with large vessel vasculitis. We use JAK inhibitors in patients with persistently elevated ISGs, although transient ISG elevation is seen in 80% of our cohort (41). Similar to IL-1 inhibition, response is dose dependent and patients may require higher doses, similar to primary interferonopathies, to achieve clinical benefit (48). Anifrolumab, an interferon α receptor (IFNAR) inhibitor, is used in some primary interferonopathies as an alternative to JAK inhibition (49, 50, 51). We have used it sporadically in patients with HA20 who have a high type I interferon signature, with favorable results. While not a current first-line treatment in our practice, further work clarifying the role of IFNAR inhibition in primary interferonopathies may change this over time.

Nearly 50% of patients develop autoimmunity, often in combination with autoinflammation, though some develop an autoimmune-predominant phenotype (e.g., antibody-mediated cytopenias, SLE-like disease, and autoimmune hepatitis; Table 1) (22). In these patients, we initiate first-line treatments per evidence-based recommendations: seropositive rheumatoid arthritis with methotrexate, class V lupus nephritis with mycophenolate and a calcineurin inhibitor, and autoimmune hepatitis with azathioprine. Many patients do not respond to these standard therapies, and we frequently switch to or add biologic DMARDs for these cases. Patients with lupus-like disease and inflammatory liver disease are frequently treated with JAK inhibitors, as these features have been associated with ISG elevation and responsiveness to JAK inhibition in our cohort (41). TNF inhibition is another commonly used strategy.

When combination therapy is necessary, our most common approach is to add a conventional DMARD, which has been a safe and effective strategy for many rheumatic diseases (52). Colchicine and PDE4 inhibitors can be added for breakthrough mucosal disease, hydroxychloroquine or topical JAK inhibitors for breakthrough cutaneous disease, methotrexate for inflammatory peripheral arthritis, and azathioprine for breakthrough inflammatory liver disease. We also use azathioprine, calcineurin inhibitors, or sirolimus for refractory immune-mediated cytopenias and adjunctive azathioprine, calcineurin inhibitors, or mycophenolate for lupus-like nephritis. Thalidomide remains an option used at other centers and in other countries (21, 23, 53). Patients with humoral immunodeficiency and CVID-like disease are treated with immunoglobulin replacement therapy in combination with conventional or biologic DMARDs.

These strategies may be ineffective in patients with severe inflammatory disease (e.g., multi-organ damage or persistent inflammation refractory to single-agent therapy). In such cases, we have used combination biologics, a strategy that has also been reported for refractory IBD and other autoinflammatory diseases (54, 55, 56). For severe autoinflammation with IBD, we have combined TNF inhibitors with IL-1 inhibitors. For persistent autoinflammation with type 1 ISG elevation or elevation of type 1/2 cytokines, we have combined IL-1 inhibition with JAK inhibition or IFNAR blockade. In one severe and refractory case, we have referred a patient for allogeneic stem cell transplantation, who has been in drug-free remission for over 2 years after transplant.

Disease flares are generally treated with short courses of corticosteroids. Patients responsive to IL-1–targeted therapies can alternatively use short courses of anakinra as needed for flares. In patients with a history of postvaccination flares, a brief course of anakinra (or, less commonly, low-dose corticosteroids) prior to vaccination may help reduce flare risk. Anakinra and other IL-1 blockers do not affect vaccine responses, and this strategy is employed to prevent postvaccination flare in other patients with SAIDs (57, 58). Most patients do not experience postvaccination flares, and most such flares are mild; we therefore recommend against empiric anakinra or corticosteroids in patients without a prior history.

During disease flares triggered by infection or sepsis, we do not universally hold immunosuppression but rather assess the risks and benefits of holding vs. continuing immunomodulatory therapy. For example, anakinra can be continued safely even during septic shock if the underlying infection is also being treated; indeed, IL-1R inhibition has consistently demonstrated safety and even mortality benefit in sepsis patients with a hyperinflammatory phenotype (59, 60, 61). Moreover, withholding anakinra can trigger a profound rebound effect due to the drug’s short half-life, which can result in severe autoinflammation or even cytokine storm syndrome. Conversely, long-acting TNF inhibitors might be held briefly without substantial risk of harm. Similar considerations apply to surgical procedures, where the risks and benefits of withholding immunosuppression must be carefully balanced, accounting for the half-life and immunosuppressive potency of the specific therapy, the severity of the patient’s underlying disease, and the invasiveness of the procedure. As in other rheumatic diseases, the threshold for holding immunosuppression should be high in patients with organ-threatening or life-threatening disease (62). If immunosuppression must be withheld, patients should be monitored closely for the development of disease flares, including cytokine storm. Empiric high-dose IL-1R inhibition, pulse dose corticosteroids, and/or interferon-blocking therapies may be warranted in these situations.

## Family testing and referral, “asymptomatic” family members

Because HA20 is highly penetrant with autosomal-dominant inheritance, asymptomatic family members confirmed or suspected to have a variant should undergo a comprehensive rheumatologic and immunologic evaluation, with consideration of genetic testing. Children of affected individuals have a 50% chance of inheriting the variant, and genetic testing may be offered prior to symptom onset: newborns and younger children may be asymptomatic until late childhood but may nonetheless be at risk of developing severe disease. For family members with subclinical or clinical features, treatment should be strongly considered once molecular genetic testing confirms the diagnosis. Patients with asymptomatic or preclinical disease should be screened periodically, with a particular emphasis on mild yet clinically relevant disease manifestations and on lymphoma risk, especially in adults. All patients with known or suspected HA20 should be educated about both the classic features and the heterogeneous nature of the disease. Pregnancy and conception counseling should be provided to affected individuals of reproductive age.

## Conclusions

HA20 is an underrecognized, highly heterogeneous PIRD with features of autoinflammation, autoimmunity, atopy, lymphoproliferation, and immunodeficiency. Clinicians should maintain a high suspicion for HA20 when evaluating patients with a personal and/or family history of early-onset BD, IBD, autoinflammation, and/or autoimmunity. HA20 should not be excluded simply because BD-like features are absent, as other phenotypes exist. Genetic testing for a pathogenic *TNFAIP3* variant is diagnostic, and current methods capture ∼95% of loss-of-function variants. Clinical evaluations should be comprehensive, screening for common and rare morbidities affecting multiple organ systems, and management requires multispecialty collaboration. Treatment should be offered to all patients, including those with mild disease. Therapies targeting A20-suppressed inflammatory pathways should be prioritized: IL-1, TNF, and/or JAK inhibitors are the treatments of choice for severely affected patients. Inhibition and response are dose-dependent and can be guided by clinical symptoms and biomarkers; higher than normal doses may be required to achieve remission. Extreme caution should be used if holding treatment during acute infection or surgery due to the high risk of disease flare. Family members should be offered genetic testing, and patients with asymptomatic or subclinical disease should still undergo periodic screening.

### Online supplemental material

Table S1 shows treatments reported in literature (non-exhaustive).

## Supplementary Material

Table S1 shows treatments reported in literature (non-exhaustive).
