# Supplementary material for: How I Treat: Haploinsufficiency of A20
Source: J Hum Immun. 2026 May 4;2(4):e20250138. doi: 10.70962/jhi.20250138 (PMC13177427; doi:10.70962/jhi.20250138)
Supplement: Table S1 — shows treatments reported in literature (non-exhaustive). [file jhi_20250138_tables1.docx]

**Supplementary Table 1: Treatments reported in literature (non-exhaustive)**

| Treatments | Karri 2024  (Karri et al., 2024)  n = 189 | Shiraki 2025  (Shiraki et al., 2025)  n = 54 | Others 9  (Xue et al., 2025; Mastrolia et al., 2025; Shirai et al., 2024; Jayaraman and Balmuri, 2025; Potjewijd et al., 2025) | Multicenter cohort, Initial treatment post-genetic diagnosis (He et al.) |
| --- | --- | --- | --- | --- |
| CS | 48% | 70%, 38/54 | 25% | 55.2% |
| Colchicine | 26% | 69%, 37/54 | 25% | 16.4% |
| PDE4 inhibitor | NR | NR | NR | 1.5% |
| AZA | 13% | 7%, 4/54 | NR | 5.2% |
| MTX | 12% | 26%, 14/54 | 17% | 8.2% |
| Thalidomide | 9.5% | 4%, 2/54 | 33% | 26.7% |
| IL1 or IL-1R inhibitor | 7.9% | 2%, 1/54 | 17% | 16.7% |
| TNF inhibitor | 29% | 63%, 34/54? | 50% | 35.1% |
| JAK inhibitor | 6.9% | 2%, 1/54 | NR | 6.0% |
| IVIG | 7.4% | NR | NR | 2.2% |
| Other biologics | NR | NR | NR | 0.7-2.2%* |
| Other DMARDs | NR | NR | NR | 0.7-11.2%** |

*varies for different biologics; **varies for different DMARDs

Abbreviations: CS, corticosteroids; PDE4, phosphodiesterase 4; AZA, azathioprine; MTX, methotrexate; IL-1, interleukin-1; IL-1R, interleukin-1 receptor; TNF, tumor necrosis factor; JAK, Janus kinase; IVIG, intravenous immunoglobulin; DMARD, disease modifying anti-rheumatic drug.
